# Pattern variation is linked to anti-predator coloration in butterfly larvae

**DOI:** 10.1098/rspb.2023.0811

**Published:** 2023-06-28

**Authors:** Callum F. McLellan, Innes C. Cuthill, Stephen H. Montgomery

**Affiliations:** School of Biological Sciences, University of Bristol, 24 Tyndall Avenue, Bristol BS8 1TQ, UK

**Keywords:** patterning, aposematism, crypsis, camouflage, defensive coloration, gregariousness

## Abstract

Prey animals typically try to avoid being detected and/or advertise to would-be predators that they should be avoided. Both anti-predator strategies primarily rely on colour to succeed, but the specific patterning used is also important. While the role of patterning in camouflage is relatively clear, the design features of aposematic patterns are less well understood. Here, we use a comparative approach to investigate how pattern use varies across a phylogeny of 268 species of cryptic and aposematic butterfly larvae, which also vary in social behaviour. We find that longitudinal stripes are used more frequently by cryptic larvae, and that patterns putatively linked to crypsis are more likely to be used by solitary larvae. By contrast, aposematic larvae are more likely to use horizontal bands and spots, but we find no differences in the use of individual pattern elements between solitary and gregarious aposematic species. However, solitary aposematic larvae are more likely to display multiple pattern elements, whereas those with no pattern are more likely to be gregarious. Our study advances our understanding of how pattern variation, coloration and social behaviour covary across lepidopteran larvae, and highlights new questions about how patterning affects larval detectability and predator responses to aposematic prey.

## 1. Introduction

Many organisms that are prey to visual hunters rely on coloration to avoid being eaten [1–3]. For example, cryptic prey must closely match the colours of their background, whereas aposematic prey typically use colours that stand out from theirs, or at least distinguish them from palatable cryptic prey [4,5]. Prey also often use multiple different colours in a species-specific stereotypical pattern as part of their anti-predator appearance. However, the specific elements that these patterns consist of, and how they might vary between strategies, has received less attention [3].

A pattern consisting of multiple different colours can be necessary for cryptic prey to better match the many different shades and hues of their background [5–7], but this is not the only way patterning is used to avoid detection. Prey backgrounds might also contain distinct, frequently occurring textures or patterns, the appearance of which prey can match with their own pattern to reduce detectability [7]. A pattern containing patches of contrasting colour can also reduce the ability of predators to detect the outline of prey by visually disrupting the organism's edges [8,9]. By contrast, for aposematic species, the precise benefits of pattern use, instead of a single, conspicuous colour, are less obvious, given that colour is thought to be the more salient element of warning signals for birds [10–12]. Nevertheless, aposematic signals often contain multiple contrasting colours with repeating pattern elements [5,13]. Evidence suggests that these patterns help to enhance warning signal conspicuousness [14], aversiveness or recognizability/memorability [15–19], distinctiveness from more profitable prey [18,20], and may also reduce long-distance detectability of prey [21,22]. Yet, despite the putative benefits that patterns grant aposematic prey, few studies have looked for potential variation in these benefits between specific patterns.

By determining how patterning varies across cryptic and aposematic prey, it may be possible to understand how certain patterns are better suited to specific roles. For example, patterns that match a texture commonly found in the environment should be suitable for crypsis [23,24], such as longitudinal stripes in a grassy habitat [13,25,26]. Contrasting, peripherally placed, patches of colour may also be especially important in crypsis when used for a disruptive effect [8,27,28]. Similarly, countershading, the contrast of dark dorsal coloration with a lighter ventral colour, can act as camouflage in more than one way [29–31]. Conversely, common patterns used by aposematic prey include spots and transverse bands of highly contrasting colour, such as those seen in ladybirds [32], aculeate hymenoptera [33], frogs [26,34] and snakes [35,36].

Prey also gain survival benefits by aggregating, as this reduces their individual chances of being singled-out by a predator. Individual predation risk is further minimized when prey discourage predators using aposematism [37–40]. Additionally, grouping is believed to collectively enhance the warning signal of some species, making predators less likely to attack [37,39,41–43]. It is therefore possible that certain patterns are more aversive to predators than others when displayed as a group, and we should expect such patterns to have evolved in aggregated species more often than solitary [43].

Finally, the number of distinct elements comprising prey patterns might affect how the overall pattern is used in either anti-predator strategy. For example, a multi-element pattern might be detrimental for cryptic prey positioned on largely homogeneous backgrounds, such as leaves or stems, when only one element is more suitable for a disruptive or background-matching pattern. Simpler aposematic patterns, consisting of one or two elements, might be easier for predators to remember and recognize [44]. Alternatively, multi-element warning signals may be needed to maximize distinctiveness from more profitable prey or reduce overall detectability [45].

Here, we use larval butterflies as a model system to test hypotheses regarding variable patterning of prey under strong selection from mainly visual predators [46]. We focus on larval butterflies because of their wide variation in anti-predator coloration and behaviour [40], making them ideal candidates for comparative study. Importantly, unlike studies of patterning in mature animals, we can also be confident that larval patterning is not involved in sexual signalling; indeed, given the low acuity of caterpillar eyes, any type of intra-specific visual signalling is unlikely. Using a dataset of 268 species distributed evenly across the majority of butterfly clades, we investigate whether specific patterns are used more frequently by either cryptic or aposematic larvae, whether larval social behaviour influences this pattern use and how pattern element number within displays is linked to these morphological and behavioural traits.

## 2. Methods

### (a) Trait data

**Figure 1.**
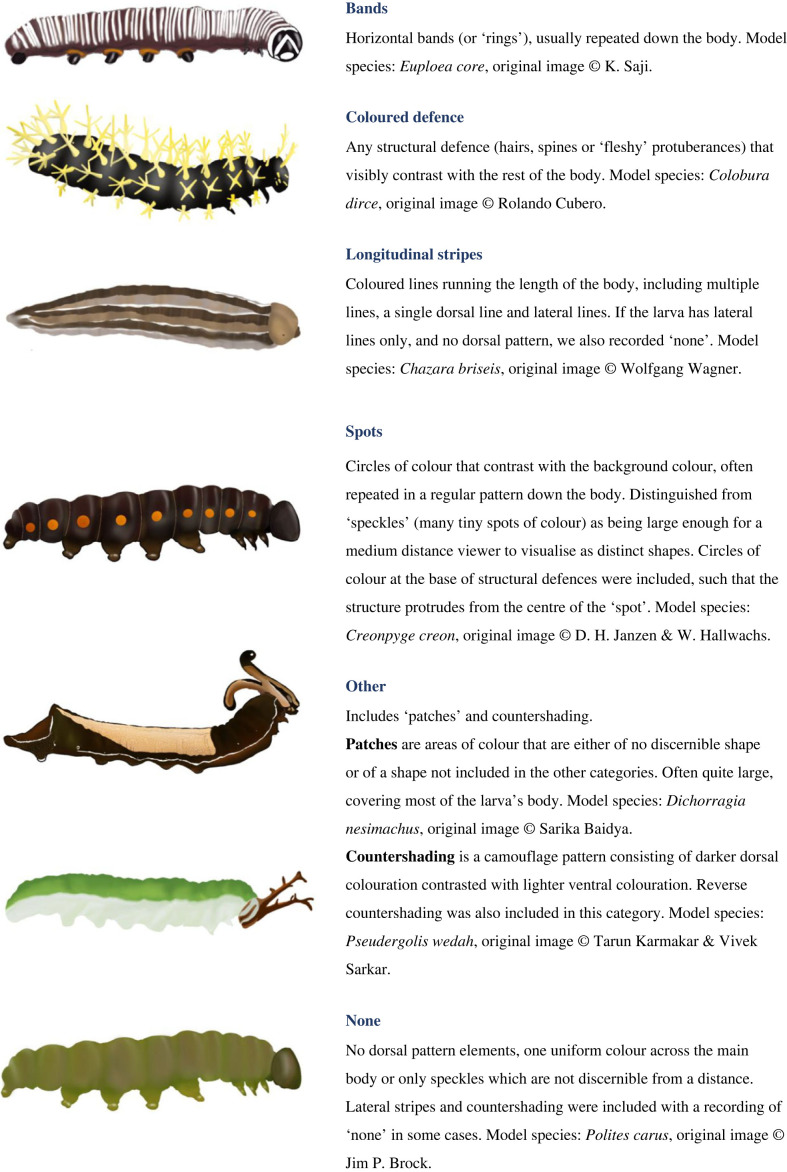
Descriptions and visual examples of the larval pattern elements considered in our study. Illustrations are based on images of real species, but have been ‘adapted’ so that only one pattern element is shown. The species that illustrations are based on, along with the original image credit, are provided in each section. Illustrations by Amaia Alcalde.

We collated data on larval social behaviour, colour strategy and structural defence presence collected by McLellan *et al*. [40] based on the published butterfly phylogeny from Chazot *et al*. [47]. Originally, these data were collected from reports on larval phenotype in the literature and images of larvae from trusted online sources [40]. This resulted in us sampling a minority of all butterfly species, but provides a phylogenetically and ecologically unbiased sampling method, with higher sampling density than existing pan-lepidopteran datasets. We searched for images of each larval species in the McLellan *et al*. dataset using three main online sources [48–50] and several online identification repositories (details of these sources, including their reliability, are given in the electronic supplementary material), including some extra species which were not in this original dataset but are included in Chazot *et al*.'s phylogeny. From the images, we recorded the pattern elements used by the late/final stage larva. For gregarious species, we used images of the latest instar available while still showing gregarious behaviour. We discounted images of putative first instars as larvae are unlikely to have developed their colour pattern at this stage [51–53]. Patterns were recorded from images by a single researcher. The pattern elements recorded were bands, coloured defences, longitudinal stripes, ‘other’ (includes patches and countershading), spots and no pattern element (detailed descriptions provided in figure 1). A recent study by Robinson *et al*. [13] independently devised a highly similar categorization of larval colour strategy and patterning, in which specific colour combinations, and whether colours typically contrast with the background or not, were used to infer crypsis and aposematism. Nevertheless, we recognize that pattern categorization, and whether a signal is perceived as aversive or not, may be dependent on the natural setting and distance of the viewer. In total, we recorded 284 pattern elements across 268 butterfly genera, with each genus in the phylogeny represented by a single species (electronic supplementary material, figure S1, see electronic supplementary material, methods for further details).

### (b) Phylogenetic analyses

Using the phylogenetic tree from Chazot *et al*. [47], pruned to remove species with missing data, we first calculated the phylogenetic signal of each trait as estimated by Pagel's *λ* [54]. With data formatted in ‘wide’ layout (one row per species, a column per pattern element and binary 0/1 if they exhibited each one), we used MCMCglmms [55] to test for correlated evolution between pattern elements, and between pattern elements and the two behavioural categories (solitary/gregarious) while controlling for the influence of phylogenetic relatedness. We ran MCMC models for 5.1 million iterations, including a 0.1 million burn-in, and sample storage frequency of every 500 iterations, with significance of the model calculated as the probability of the parameter value being different from zero (*pMCMC*). We also report each model cofactor's posterior mean (p-mean) and its 95% confidence intervals (CI). We also examined whether the number of different pattern elements (excluding entries for ‘none’) each species uses (categorical: 0 = no pattern, 1 = one pattern element, 2 = more than one element) was associated with larval colour strategy to test the hypothesis that cryptic patterns require fewer elements to provide effective camouflage. We then tested both colour strategies separately to see if any category of pattern element number is used most by each.

## 3. Results

### (a) Colour patterns are evolutionarily labile and co-occurrence is non-random

**Figure 2.**
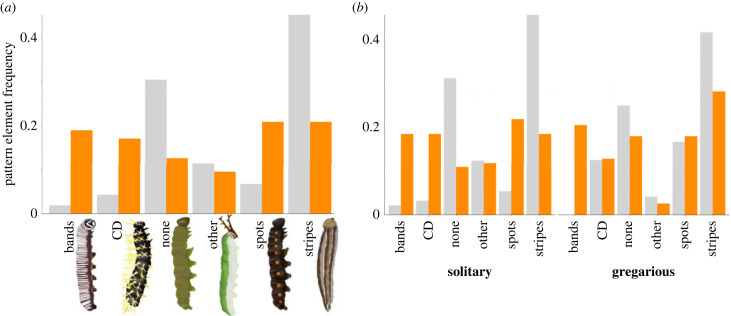
The frequency of pattern element use by 268 larval butterfly species separated by their anti-predator colour strategy (grey = cryptic larvae, orange = aposematic larvae, CD = coloured defence). (*a*) Overall pattern element use across both social behaviours. (*b*) Pattern element use separated by their social behaviour.

Both larval social behaviour and colour strategy show strong phylogenetic signals (table 1). By contrast, the majority of the pattern elements appear more evolutionarily labile, with only longitudinal stripes having a strong phylogenetic signal (table 1). Longitudinal stripes are also the most frequently exhibited pattern element across all larvae in our dataset (figure 2*a*). Both bands and longitudinal stripes have significant, negative interactions with most of the other pattern elements (electronic supplementary material, table S2), meaning that when one element is present, the other is not. Whereas a coloured structural defence and no pattern are the only two categories with a significant, positive interaction (electronic supplementary material, table S2), meaning they often co-occur. The ‘other’ category of pattern element is the only category that does not have a significant, negative interaction with the ‘none’ (no pattern) category (electronic supplementary material, table S2), most likely because of the inclusion of countershading in the former category.

**Table 1.** Phylogenetic signal (represented by Pagel's lambda) of larval traits (CD = coloured defence). Asterisks denote lambda values that are significantly different from one, indicating phylogenetic independence.

trait	lambda	loglik		LRstat	*p*-value
		null	lambda		
social behaviour	0.944	−135.927	−135.560	0.733	0.392
colour strategy	0.950	−132.058	−131.489	1.138	0.286
bands	0.321	−109.490	−101.271	16.437	< 0.001*
CD	0.548	−64.780	−60.719	8.123	0.004*
none	0.365	−173.408	−162.919	20.979	< 0.001*
other	0.095	−118.362	−111.014	14.696	< 0.001*
spots	0.410	−128.192	−123.985	8.414	0.004*
stripes	0.871	−174.270	−172.429	3.682	0.055
pattern element number	0.513	−257.421	−245.810	23.222	< 0.001*

### (b) Aposematic and cryptic species differ in their pattern use

After accounting for phylogenetic relatedness, only bands and spots are used more frequently by aposematic species (bands: p-mean = 127.96, 95% CI 21.49–254.56, *pMCMC* < 0.001; spots: p-mean = 51.83, 95% CI –3.45–131.37, *pMCMC* = 0.048; table 2), whereas longitudinal stripes are used more frequently by cryptic species (p-mean = −52.113, 95% CI −127.796– 0.954, *pMCMC* = 0.025; table 2). Additionally, larvae which use multiple elements in their patterns are more likely to be aposematic than cryptic (p-mean = 118.35, 95% CI 10.07–258.22, *pMCMC* = 0.017; electronic supplementary material, table S3).

**Table 2.** Results from MCMCglmms looking at interactions between larval colour strategy and pattern element, not accounting for social behaviour. Shown are the posterior mean (p-mean) and 95% confidence intervals (CI) of models. Coloured defence (CD) model *n* = 101, all other models *n* = 268. Significant interactions are denoted by an asterisk.

pattern element	p-mean	95% CI		*_P_MCMC*
		lower	upper	
bands	127.96	21.49	254.56	<0.001*
CD	13.55	−73.41	100.30	0.702
none	−40.98	−113.45	11.00	0.095
other	−8.775	−93.072	67.562	0.816
spots	51.83	−3.45	131.37	0.048*
stripes	−52.113	−127.796	0.954	0.025*

### (c) Cryptic species are more constrained in pattern use, while aposematic species are more patterned when solitary

As previously reported [40], we found that gregarious larvae are more likely to be aposematic than cryptic (p-mean = 59.087, 95% CI 1.237–177.903, *pMCMC* = 0.007). Our three-factor analysis shows that crypsis coupled with either longitudinal stripes, patches and/or countershading, or no pattern, are strong predictors of solitariness in larvae (table 3). However, there are no interactions between aposematism and the pattern elements that predict either social behaviour (table 3). Finally, we found no evidence of a relationship between larval social behaviour and pattern element number (electronic supplementary material, table S3). Additionally, our model revealed no interactions between crypsis and numbers of pattern elements used which predict larval social behaviour (table 4). However, aposematic larvae with no pattern or a single pattern element are more likely to be gregarious than solitary (table 4), whereas aposematic larvae with multiple pattern elements are more likely to be solitary (p-mean = −302.850, 95% CI −636.690 to −32.994, *pMCMC* = 0.011; table 4).

**Table 3.** Three-factor analysis between larval colour strategy and pattern elements (CD = coloured defence) against social behaviour. Significant interactions are denoted by an asterisk.

colour strategy	pattern element	p-mean	95% CI		*_P_MCMC*
			lower	upper	
crypsis	bands	−2743.229	−6260.236	215.320	0.064
	CD	−216.824	−509.804	66.019	0.135
	none	−295.785	−551.416	−63.940	0.011*
	other	−350.442	−729.095	−37.356	0.022*
	spots	−192.537	−458.749	66.174	0.131
	stripes	−214.534	−434.007	−6.882	0.035*
aposematism	bands	−28.314	−188.588	133.655	0.721
	CD	−116.264	−336.020	61.637	0.208
	none	−83.291	−280.816	101.247	0.375
	other	−206.731	−595.622	98.653	0.179
	spots	−64.502	−254.413	79.484	0.412
	stripes	−325.018	−761.768	97.561	0.109

**Table 4.** Three-factor analysis between larval social behaviour, colour and the number of pattern elements used. Significant interactions are denoted by an asterisk.

trait	pattern element number	p-mean	95% CI		*_P_MCMC*
			lower	upper	
crypsis	0	−72.289	−290.309	121.394	0.462
	1	−91.250	−258.660	53.670	0.200
	>1	128.690	−62.684	334.190	0.149
aposematism	0	375.334	108.087	656.003	0.004*
	1	119.992	−6.951	270.636	0.043*
	>1	−302.850	−636.690	−32.994	0.011*

## 4. Discussion

To our knowledge, our study provides the first investigation into the covariance of colour pattern and grouping behaviour in lepidopteran larvae. Indeed, to date, only one other study has explored the pattern variation between lepidopteran larvae using different anti-predator colour strategies [13]. Our data reveal that both cryptic and aposematic anti-predator colour strategies each favour the use of one pattern in particular across butterfly larvae. We also show that patterns displayed with crypsis, but not aposematism, predict larval social behaviour. While aposematism and gregarious behaviour are strongly linked overall, we found no evidence of selective pattern use in aposematic, gregarious species. However, we show that there is behaviour-based variation in aposematic pattern element number, where multi-element patterns are more likely to be seen in solitary species, and gregarious species are more likely to lack patterning entirely. Below, we offer explanations for these findings based on the advantages of each colour strategy and the roles patterning is likely to fill, with a focus on predator responses to prey appearance.

We found that the social behaviour and colour strategy of larvae are largely dependent on their recent ancestors' phenotype, whereas the evolution of most of their pattern elements is near-randomly distributed across the phylogeny. However, the comparatively high conservation across lineages of the longitudinal stripes pattern, along with its frequent use by cryptic species (figure 2*a*), suggests that it is especially useful for camouflage. This conclusion supports findings elsewhere in the literature, that longitudinal stripes provide camouflage for a number of different taxa [13,35,56–58]. Similar patterns are reported in non-venomous reptiles, where stripes are thought to aid with rapid escapes upon the detection by a predator [35,59–61]. This is unlikely to be their purpose in relatively slow-moving caterpillars however. A more likely explanation is that this pattern offers some form of camouflage to prevent initial detection. Stripes may help to hide larvae via surface disruption [62], or by matching some common aspect of the environment [13,56,57]. For background-matching camouflage to be effective, prey appearance should closely resemble both the colour and pattern of the background [24]. Longitudinal stripes are likely to be used for the latter, given that, because they are folivores, caterpillars’ backgrounds mostly consist of stems and leaf veins. Furthermore, cryptic prey on backgrounds with oriented features need to align their pattern to blend in [63,64]. Longitudinal stripes will also help in this regard, as larvae need simply to sit parallel to plant stems or grass blades' growth direction to achieve this effect. For example, many members of the Hesperiidae family, where solitariness and stripes frequently co-occur (electronic supplementary material, figure S1), are grass-feeders [40]. This environment is likely to select for a longitudinal stripes pattern and solitary lifestyle to avoid detection, as found by a recent study of larval patterning and host plant preference [13]. The benefit to camouflage of longitudinal stripes is likely why they are often displayed without any other patterns (electronic supplementary material, table S2). If stripes are mainly used to match aspects of the background, this effect may be diminished by the presence of other pattern elements.

We found that crypsis, and some of the patterns shown to be associated with crypsis (e.g. [13]), are linked to solitariness in larvae. Overall, selection should strongly favour solitariness in cryptic larvae, given that grouping necessarily increases detectability and the risk of predation [40,65]. Most larvae carrying no pattern, stripes, patches and/or countershading are cryptic (figure 2*b*), and solitariness is a way of further minimizing detectability [40]. Therefore, just as longitudinal stripes are often displayed on their own, larvae with these cryptic patterns may be solitary if their camouflaging effects are diminished by the presence of neighbours.

For aposematic species, our findings support those of a recent study using a similar approach [13], in that larvae are most likely to use a pattern of colour-contrasting, horizontal bands or spots. This suggests there is some inherent quality (or qualities) of these patterns which support effective aposematic signalling. This might be anything from distinctiveness from more profitable prey, greater aversiveness to naïve predators [16,66,67], high recognizability, memorability or conspicuousness. Aposematic prey must appear distinct from cryptic, profitable prey to reduce recognition errors made by predators [18,20,44,68,69]. Thus, because relatively few cryptic larvae use bands or spots (figure 2*a*), it is possible that these are selected as being most distinct from cryptic larvae's patterning. Colour-contrasting bands, compared to no pattern, can also increase the speed of avoidance learning by avian predators [16,19]. Alternatively, bands may simply be the most developmentally efficient way to pattern the body to include two highly contrasting colours that are also conspicuous against the background, both of which are important for predator avoidance learning [14,18,67,68,70,71]. This might explain why, like longitudinal stripes, horizontal bands are often displayed without accompanying patterns (electronic supplementary material, table S2), if bands alone are sufficient as a memorable warning signal [16,19]. Conversely, horizontal bands might have the additional benefit of *reducing* signal conspicuousness for long-range viewers, while maintaining an aversive and memorable signal for near viewers. This is the idea that the separate bands comprising a warning signal become too difficult to distinguish for a long-range viewer, and the average colour produced from this ‘blending’ effect is a close match to that of the background [22]. This distance-dependent camouflage is important to, and present in, several aposematic larvae [2,21,22], although whether its effect varies with the pattern used requires investigation.

Previous work has found that bands may be linked to sociality across various taxa [43] yet, despite the potential enhancement of the warning signal by grouping overall [13,37,41,42], we found no evidence that specific aposematic patterns are used more by solitary or gregarious larvae. This is aligned with previous findings that the patterns of warning signals are of secondary importance to avian predators, which instead focus primarily on colour [10–12]. However, we also found that larvae with mutli-element patterns are likely to be aposematic, and that multi-element aposematic patterns are more often displayed by solitary larvae. Aposematic larvae might use more pattern elements if this increases their distinction from the background and/or from more profitable prey, thus helping to reduce predator recognition errors [18,20,44,68–70,72]. If multi-element signals have this effect, it is likely to be disproportionately more important to solitary larvae, as gregarious larvae are inherently more conspicuous and better protected, via dilution, from predation (e.g. [40]). Furthermore, multi-element patterns might help to reduce the overall detectability of solitary prey depending on visual noise in the environment (e.g. [45]), but have less of an effect on grouped, conspicuous larvae. Our findings do not appear to support the hypothesis that aposematic signals consisting of fewer pattern elements are favoured in solitary larvae because they enhance avoidance learning [44], thus predator responses to prey pattern complexity require proper investigation.

The behaviour-based imbalance in predation risk between aposematic larvae may also explain our finding that aposematic larvae with no or one pattern element are more likely to be gregarious than solitary. Solitary, aposematic larvae only benefit from their warning signal if predators recognize and avoid them based on previous experience [73], whereas gregarious larvae are unlikely to be attacked immediately after a predator samples one of their neighbours [18,42]. Therefore, it is more important for solitary larvae to present a conspicuous, distinct and/or memorable signal [68,69]. To have these properties, this signal is unlikely to be a single, uniform colour in most systems [14,16,19,44]. For example, our data suggest that both horizontal bands and spots are selected for use in aposematism (table 2), but a lack of pattern is not. Therefore, it is likely that solitary larvae need at least one pattern for their signal to confer effective anti-predator protection, whereas grouped larvae only need to show that they are as unprofitable as their neighbour (e.g. [74]), and so may not need particularly memorable or aversive signals. Furthermore, larvae with a single pattern element might appear to a predator as displaying a multi-element pattern when grouped, whereas a similar appearance can only be achieved for solitary larvae with an individual, multi-element pattern. However, it is currently unclear whether a multi-element aposematic pattern (or the appearance of one) confers greater anti-predator benefits than a simpler one.

Finally, selection against multi-element patterns in gregarious larvae may be especially strong if there are physiological costs associated with their production [75–77]. Aposematism is believed to have preceded the evolution of larval gregariousness in most cases [40,78], and so there may be a drive to reduce pattern element number once larvae have become gregarious. Pigment production costs might also explain our finding that larvae's contrasting, and often conspicuously, coloured structural defences are frequently accompanied by a lack of patterning across their body. This suggests that coloured defences are sometimes used as the warning signal, rather than specific patterning across the body. It may be that simply highlighting the presence of their structural defences may lessen the need for larvae to otherwise advertise their toxicity.

Our study is one of the first to highlight how pattern use varies across both cryptic and aposematic larvae. We note that there are likely to be aspects of prey pattern use that we could not account for in the present study, including exact details of how they are perceived by the predators which they have evolved in response to. For example, we did not account for interactions between variations in the size of the prey or pattern elements, which might influence the effect they have on predators [79]. Nevertheless, we have identified broad distinctions in pattern use between cryptic and aposematic larvae, and brought to light several novel questions deserving study. In particular, we highlight the frequent appearance of longitudinal stripes among cryptic caterpillars, which indicates that this may be the most effective camouflaging pattern for larvae given their background. We show that colour-contrasting bands and spots are frequently used in aposematism, but the properties which favour their use in warning signalling are unclear. Finally, we identify gaps in our understanding regarding pattern element number in larval displays, such as whether multi-element patterns make better warning signals, particularly for solitary larvae. Furthermore, does selection favour the use of fewer pattern elements in grouped, aposematic larvae, or is there simply no strong selection driving pattern element number instead? We believe that each of these questions present interesting avenues for further study.

## Data Availability

Data available from the Dryad Digital Repository: https://doi.org/10.5061/dryad.3xsj3txmn [80]. Additional information is provided in the electronic supplementary material [81].

## References

[1] Caro T, Sherratt TN, Stevens M. 2016 The ecology of multiple colour defences. Evol. Ecol. **30**, 797-809. (10.1007/s10682-016-9854-3)

[2] Cuthill IC et al. 2017 The biology of color. Science **357**, eaan0221. (10.1126/science.aan0221) 28774901

[3] Caro T, Koneru M. 2021 Towards an ecology of protective coloration. Biol. Rev. **96**, 611-641. (10.1111/brv.12670) 33258554

[4] Merilaita S, Ruxton GD. 2007 Aposematic signals and the relationship between conspicuousness and distinctiveness. J. Theor. Biol. **245**, 268-277. (10.1016/j.jtbi.2006.10.022) 17157321

[5] Ruxton GD, Allen WL, Sherratt TN, Speed MP. 2019 Avoiding attack: the evolutionary ecology of crypsis, aposematism, and mimicry. Oxford, UK: Oxford University Press.

[6] Merilaita S, Stevens M. 2011 Crypsis through background matching. In Animal camouflage: mechanisms and function (eds M Stevens, S. Merilaita), pp. 17-33. Cambridge, UK: Cambridge University Press.

[7] Cuthill IC. 2019 Camouflage. J. Zool. **308**, 75-92. (10.1111/jzo.12682)

[8] Cuthill IC, Stevens M, Sheppard J, Maddocks T, Párraga CA, Troscianko TS. 2005 Disruptive coloration and background pattern matching. Nature **434**, 72-74. (10.1038/nature03312) 15744301

[9] Stevens M, Cuthill IC. 2006 Disruptive coloration, crypsis and edge detection in early visual processing. Proc. R. Soc. B **273**, 2141-2147. (10.1098/rspb.2006.3556) PMC1635512 16901833

[10] Aronsson M, Gamberale-Stille G. 2008 Domestic chicks primarily attend to colour, not pattern, when learning an aposematic coloration. Anim. Behav. **75**, 417-423. (10.1016/j.anbehav.2007.05.006)

[11] Kazemi B, Gamberale-Stille G, Tullberg BS, Leimar O. 2014 Stimulus salience as an explanation for imperfect mimicry. Curr. Biol. **24**, 965-969. (10.1016/j.cub.2014.02.061) 24726157

[12] Rönkä K, De Pasqual C, Mappes J, Gordon S, Rojas B. 2018 Colour alone matters: no predator generalization among morphs of an aposematic moth. Anim. Behav. **135**, 153-163. (10.1016/j.anbehav.2017.11.015)

[13] Robinson ML, Weber MG, Freedman MG, Jordan E, Ashlock SR, Yonenaga J, Strauss SY. 2023 Macroevolution of protective coloration across caterpillars reflects relationships with host plants. Proc. R. Soc. B **290**, 20222293. (10.1098/rspb.2022.2293) PMC9845978 36651051

[14] Osorio D, Jones CD, Vorobyev M. 1999 Accurate memory for colour but not pattern contrast in chicks. Curr. Biol. **9**, 199-202. (10.1016/S0960-9822(99)80089-X) 10074430

[15] Kauppinen J, Mappes J. 2003 Why are wasps so intimidating: field experiments on hunting dragonflies (Odonata: *Aeshna grandis*). Anim. Behav. **66**, 505-511. (10.1006/anbe.2003.2225)

[16] Hauglund K, Hagen SB, Lampe HM. 2006 Responses of domestic chicks (*Gallus gallus domesticus*) to multimodal aposematic signals. Behav. Ecol. **17**, 392-398. (10.1093/beheco/arj038)

[17] Prudic KL, Skemp AK, Papaj DR. 2007 Aposematic coloration, luminance contrast, and the benefits of conspicuousness. Behav. Ecol. **18**, 41-46. (10.1093/beheco/arl046)

[18] Joron M. 2009 Aposematic coloration. In Encyclopedia of insects (eds VH Resh, RT Cardé), pp. 33-38. New York, NY: Academic Press.

[19] Aronsson M, Gamberale-Stille G. 2013 Evidence of signaling benefits to contrasting internal color boundaries in warning coloration. Behav. Ecol. **24**, 349-354. (10.1093/beheco/ars170)

[20] Sherratt TN, Beatty CD. 2003 The evolution of warning signals as reliable indicators of prey defense. Am. Nat. **162**, 377-389. (10.1086/378047) 14582002

[21] Tullberg BS, Merilaita S, Wiklund C. 2005 Aposematism and crypsis combined as a result of distance dependence: functional versatility of the colour pattern in the swallowtail butterfly larva. Proc. R. Soc. B **272**, 1315-1321. (10.1098/rspb.2005.3079) PMC1560331 16006332

[22] Barnett JB et al. 2017 Stripes for warning and stripes for hiding: spatial frequency and detection distance. Behav. Ecol. **28**, 373-381. (10.1093/beheco/arw168)

[23] Endler JA. 1984 Progressive background matching in moths, and a quantitative measure of crypsis. Biol. J. Linn. Soc. **22**, 187-231. (10.1111/j.1095-8312.1984.tb01677.x)

[24] Michalis C, Scott-Samuel NE, Gibson DP, Cuthill IC. 2017 Optimal background matching camouflage. Proc. R. Soc. B **284**, 20170709. (10.1098/rspb.2017.0709) PMC5524497 28701559

[25] Godfrey D, Lythgoe JN, Rumball DA. 1987 Zebra stripes and tiger stripes: the spatial frequency distribution of the pattern compared to that of the background is significant in display and crypsis. Biol. J. Linn. Soc. **32**, 427-433. (10.1111/j.1095-8312.1987.tb00442.x)

[26] Rojas B. 2017 Behavioural, ecological, and evolutionary aspects of diversity in frog colour patterns. Biol. Rev. **92**, 1059-1080. (10.1111/brv.12269) 27020467

[27] Merilaita S. 1998 Crypsis through disruptive coloration in an isopod. Proc. R. Soc. B **265**, 1059-1064. (10.1098/rspb.1998.0399)

[28] Egan J, Sharman RJ, Scott-Brown KC, Lovell PG. 2016 Edge enhancement improves disruptive camouflage by emphasising false edges and creating pictorial relief. Sci. Rep. **6**, 1-9. (10.1038/srep38274) 27922058 PMC5138594

[29] Rowland HM, Cuthill IC, Harvey IF, Speed MP, Ruxton GD. 2008 Can't tell the caterpillars from the trees: countershading enhances survival in a woodland. Proc. R. Soc. B **275**, 2539-2545. (10.1098/rspb.2008.0812) PMC2605806 18700207

[30] Allen WL, Baddeley R, Cuthill IC, Scott-Samuel NE. 2012 A quantitative test of the predicted relationship between countershading and lighting environment. Am. Nat. **180**, 762-776. (10.1086/668011) 23149401

[31] Cuthill IC, Sanghera NS, Penacchio O, Lovell PG, Ruxton GD, Harris JM. 2016 Optimizing countershading camouflage. Proc. Natl Acad. Sci. USA **113**, 13 093-13 097. (10.1073/pnas.1611589113) PMC5135326 27807134

[32] Arenas LM, Troscianko J, Stevens M. 2014 Color contrast and stability as key elements for effective warning signals. Front. Evol. Ecol. **2**, 25. (10.3389/fevo.2014.00025)

[33] Willadsen PC. 2022 Aculeate hymenopterans as aposematic and mimetic models. Front. Evol. Ecol. **10**, 827319. (10.3389/fevo.2022.827319)

[34] Amézquita A, Castro L, Arias M, González M, Esquivel C. 2013 Field but not lab paradigms support generalisation by predators of aposematic polymorphic prey: the *Oophaga histrionica* complex. Evol. Ecol. **27**, 769-782. (10.1007/s10682-013-9635-1)

[35] Allen WL, Baddeley R, Scott-Samuel NE, Cuthill IC. 2013 The evolution and function of pattern diversity in snakes. Behav. Ecol. **24**, 1237-1250. (10.1093/beheco/art058)

[36] Davis-Rabosky AR, Cox CL, Rabosky DL, Holmes IA, Feldman A, McGuire JA. 2016 Coral snakes predict the evolution of mimicry across New World snakes. Nat. Commun. **7**, 1-9. 10.1038/ncomms11484 PMC4858746 27146100

[37] Gamberale G, Tullberg BS. 1996 Evidence for a more effective signal in aggregated aposematic prey. Anim. Behav. **52**, 597-601. (10.1006/anbe.1996.0200)

[38] Riipi M, Alatalo RV, LindstroÈm L, Mappes J. 2001 Multiple benefits of gregariousness cover detectability costs in aposematic aggregations. Nature **413**, 512-514. (10.1038/35097061) 11586357

[39] Rowland HM, Ruxton GD, Skelhorn J. 2013 Bitter taste enhances predatory biases against aggregations of prey with warning coloration. Behav. Ecol. **24**, 942-948. (10.1093/beheco/art013)

[40] McLellan CF, Cuthill IC, Montgomery SH. In press. Warning coloration, body size and the evolution of gregarious behavior in butterfly larvae. Am. Nat. **202**. (10.1086/724818) 37384762

[41] Gamberale G, Tullberg BS. 1998 Aposematism and gregariousness: the combined effect of group size and coloration on signal repellence. Proc. R. Soc. B **265**, 889-894. (10.1098/rspb.1998.0374)

[42] Svádová KH, Exnerová A, Štys P. 2014 Gregariousness as a defence strategy of moderately defended prey: experiments with *Pyrrhocoris apterus* and avian predators. Behaviour **151**, 1617-1640. (10.1163/1568539X-00003208)

[43] Negro JJ, Doña J, Blázquez MC, Rodríguez A, Herbert-Read JE, Brooke MDL. 2020 Contrasting stripes are a widespread feature of group living in birds, mammals and fishes. Proc. R. Soc. B **287**, 20202021. (10.1098/rspb.2020.2021) PMC7657865 33049169

[44] Stevens M, Ruxton GD. 2012 Linking the evolution and form of warning coloration in nature. Proc. R. Soc. B **279**, 417-426. (10.1098/rspb.2011.1932) PMC3234570 22113031

[45] Rojas B, Rautiala P, Mappes J. 2014 Differential detectability of polymorphic warning signals under varying light environments. Behav. Processes **109**, 164-172. (10.1016/j.beproc.2014.08.014) 25158931

[46] Sugiura S. 2020 Predators as drivers of insect defenses. Entomol. Sci. **23**, 316-337. (10.1111/ens.12423)

[47] Chazot N et al. 2019 Priors and posteriors in Bayesian timing of divergence analyses: the age of butterflies revisited. Syst. Biol. **68**, 797-813. (10.1093/sysbio/syz002) 30690622 PMC6893297

[48] Allen TJ, Brock JP, Glassberg J. 2005 Caterpillars in the field and garden. New York, NY: Oxford University press.

[49] Warren AD, Davis KJ, Stangeland EM, Pelham JP, Willmott KR, Grishin NV. 2016 Illustrated lists of American butterflies. See http://www.butterfliesofamerica.com.

[50] Kunte K. 2023 About us. Butterflies of India, v. 4.12. See https://www.ifoundbutterflies.org/about-us.

[51] Nylin S, Gamberale-Stille G, Tullberg BS. 2001 Ontogeny of defense and adaptive coloration in larvae of the comma butterfly, *Polygonia c-album* (Nymphalidae). J. Lepid. Soc. **55**, 69-73.

[52] Grant JB. 2007 Ontogenetic colour change and the evolution of aposematism: a case study in panic moth caterpillars. J. Anim. Ecol. **76**, 439-447. (10.1111/j.1365-2656.2007.01216.x) 17439461

[53] Sandre SL, Tammaru T, Maend T. 2007 Size-dependent colouration in larvae of *Orgyia antiqua* (Lepidoptera: Lymantriidae): a trade-off between warning effect and detectability? Eur. J. Entomol. **104**, 745-752. (10.14411/eje.2007.095)

[54] Pagel M. 1999 Inferring the historical patterns of biological evolution. Nature **401**, 877-884. (10.1038/44766) 10553904

[55] Hadfield JD. 2010 MCMC methods for multi-response generalized linear mixed models: the MCMCglmm R Package. J. Stat. Softw. **33**, 1-22. (10.18637/jss.v033.i02)20808728

[56] Sherbrooke WC. 2002 Do vertebral-line patterns in two horned lizards (*Phrynosoma spp.*) mimic plant-stem shadows and stem litter? J. Arid. Environ. **50**, 109-120. (10.1006/jare.2001.0852)

[57] Farkas TE, Mononen T, Comeault AA, Hanski I, Nosil P. 2013 Evolution of camouflage drives rapid ecological change in an insect community. Curr. Biol. **23**, 1835-1843. (10.1016/j.cub.2013.07.067) 24055155

[58] Barnett JB, Varela BJ, Jennings BJ, Lesbarrères D, Pruitt JN, Green DM. 2021 Habitat disturbance alters color contrast and the detectability of cryptic and aposematic frogs. Behav. Ecol. **32**, 814-825. (10.1093/beheco/arab032)

[59] Murali G, Kodandaramaiah U. 2016 Deceived by stripes: conspicuous patterning on vital anterior body parts can redirect predatory strikes to expendable posterior organs. R. Soc. Open Sci. **3**, 160057. (10.1098/rsos.160057) 27429765 PMC4929900

[60] Halperin T, Carmel L, Hawlena D. 2017 Movement correlates of lizards' dorsal pigmentation patterns. Funct. Ecol. **31**, 370-376. (10.1111/1365-2435.12700)

[61] Murali G, Merilaita S, Kodandaramaiah U. 2018 Grab my tail: evolution of dazzle stripes and colourful tails in lizards. Evol. Biol. **31**, 1675-1688. (10.1111/jeb.13364) 30102810

[62] Stevens M, Winney IS, Cantor A, Graham J. 2009 Outline and surface disruption in animal camouflage. Proc. R. Soc. B **276**, 781-786. (10.1098/rspb.2008.1450) PMC2660950 19019788

[63] Kang CK, Moon JY, Lee SI, Jablonski PG. 2012 Camouflage through an active choice of a resting spot and body orientation in moths. Evol. Biol. **25**, 1695-1702. (10.1111/j.1420-9101.2012.02557.x) 22775528

[64] Kang C, Stevens M, Moon JY, Lee SI, Jablonski PG. 2015 Camouflage through behavior in moths: the role of background matching and disruptive coloration. Behav. Ecol. **26**, 45-54. (10.1093/beheco/aru150)

[65] Lindstedt C, Huttunen H, Kakko M, Mappes J. 2011 Disentangling the evolution of weak warning signals: high detection risk and low production costs of chemical defences in gregarious pine sawfly larvae. Evol. Ecol. **25**, 1029-1046. (10.1007/s10682-010-9456-4)

[66] Smith SM. 1975 Innate recognition of coral snake pattern by a possible avian predator. Science **187**, 759-760. (10.1126/science.187.4178.759) 17795249

[67] Halpin CG, Penacchio O, Lovell PG, Cuthill IC, Harris JM, Skelhorn J, Rowe C. 2020 Pattern contrast influences wariness in naïve predators towards aposematic patterns. Sci. Rep. **10**, 9246. (10.1038/s41598-020-65754-y) 32514003 PMC7280217

[68] Servedio MR. 2000 The effects of predator learning, forgetting, and recognition errors on the evolution of warning coloration. Evolution **54**, 751-763. 10937250 10.1111/j.0014-3820.2000.tb00077.x

[69] Arenas LM, Stevens M. 2017 Diversity in warning coloration is easily recognized by avian predators. Evol. Biol. **30**, 1288-1302. (10.1111/jeb.13074) PMC5518184 28338250

[70] Gamberale-Stille G. 2001 Benefit by contrast: an experiment with live aposematic prey. Behav. Ecol. **12**, 768-772. (10.1093/beheco/12.6.768)

[71] Aronsson M, Gamberale-Stille G. 2009 Importance of internal pattern contrast and contrast against the background in aposematic signals. Behav. Ecol. **20**, 1356-1362. (10.1093/beheco/arp141)

[72] Guilford T. 1986 How do " warning colours" work? Conspicuousness may reduce recognition errors in experienced predators. Anim. Behav. **34**, 286-288. (10.1016/0003-3472(86)90034-5)

[73] Skelhorn J, Halpin CG, Rowe C. 2016 Learning about aposematic prey. Behav. Ecol. **27**, 955-964. (10.1093/beheco/arw009)

[74] Curley EA, Rowley HE, Speed MP. 2015 A field demonstration of the costs and benefits of group living to edible and defended prey. Biol. Lett. **11**, 20150152. (10.1098/rsbl.2015.0152) 26085497 PMC4528465

[75] Speed MP, Ruxton GD. 2007 How bright and how nasty: explaining diversity in warning signal strength. Evolution **61**, 623-635. (10.1111/j.1558-5646.2007.00054.x) 17348925

[76] Lindstedt C, Lindström L, Mappes J. 2009 Thermoregulation constrains effective warning signal expression. Evol. Int. J. Org. Evol. **63**, 469-478. (10.1111/j.1558-5646.2008.00561.x) 19154362

[77] Blount JD, Rowland HM, Mitchell C, Speed MP, Ruxton GD, Endler JA, Brower LP. 2023 The price of defence: toxins, visual signals and oxidative state in an aposematic butterfly. Proc. R. Soc. B **290**, 20222068. (10.1098/rspb.2022.2068) PMC9845971 36651049

[78] Wang L, Cornell SJ, Speed MP, Arbuckle K. 2021 Coevolution of group-living and aposematism in caterpillars: warning colouration may facilitate the evolution from group-living to solitary habits. BMC Ecol. Evol. **21**, 1-9. (10.1186/s12862-020-01734-0) 33583398 PMC7883577

[79] Preißler K, Pröhl H. 2017 The effects of background coloration and dark spots on the risk of predation in poison frog models. Evol. Ecol. **31**, 683-694. (10.1007/s10682-017-9903-6)

[80] McLellan CF, Cuthill IC, Montgomery SH. 2023 Data from: Pattern variation is linked to anti-predator colouration in butterfly larvae. Dryad Digital Repository. (10.5061/dryad.3xsj3txmn)

[81] McLellan CF, Cuthill IC, Montgomery SH. 2023 Pattern variation is linked to anti-predator colouration in butterfly larvae. Figshare. (10.6084/m9.figshare.c.6700028)

